# Can Survey Measures Predict Key Performance Indicators of Safety? Confirmatory and Exploratory Analyses of the Association Between Self-Report and Safety Outcomes in the Maritime Industry

**DOI:** 10.3389/fpsyg.2020.00976

**Published:** 2020-05-29

**Authors:** Line Raknes Hjellvik, Bjørn Sætrevik

**Affiliations:** Operational Psychology Research Group, Department of Psychosocial Science, Faculty of Psychology, University of Bergen, Bergen, Norway

**Keywords:** chemical tanker vessels, maritime safety, self-report and objective outcomes, pre-registered study, structural equation modelling

## Abstract

Safety management may be improved if managers implement measures based on reliable empirical knowledge about how psychological factors cause or prevent accidents. While such factors are often investigated with self-report measures, few studies in the maritime industry have investigated whether self-report measures predict objectively registered accidents. The current pre-registered study used structural equation modelling to test whether “Safety attitude,” “Situation awareness,” “Reporting attitude” and “Safe behaviour” predicted “Number of reports” and “Number of safety events” in the following year. The study was conducted among crew on chemical tanker vessels operating in Arctic and Baltic waters. The pre-registered model of expected associations between self-reported safety factors and recorded safety outcomes was not supported. However, an exploratory model based on the pre-registered hypotheses supported an association between self-reported “Safe behaviour” and the overall number of recorded safety outcomes. While much safety research in the maritime industry builds on the assumption that self-reported behaviour, attitude or cognitions are causally related to actual accidents, the current study shows that such a relationship can be difficult to confirm. Until more conclusive studies are performed, the assumed causal relationship between self-reported psychological factors and safety outcomes should be treated with caution.

## Introduction

Safety research in the maritime industry has described how technological and human factors may contribute in reducing risks in various work environments. Many studies have investigated human factors in the maritime industry, but investigations of the relationship between behaviour and actual accidents or accident reports are lacking (as argued by e.g., Hetherington et al., 2006). Various approaches have been applied to investigate human factors, errors, reliability and variability relevant for safety, such as the Human Factors Analysis and Classification System (Celik and Cebi, 2009; Chauvin et al., 2013), Functional Resonance Analysis Method (de Vries, 2017; Patriarca and Bergström, 2017), human reliability analysis (Akyuz and Celik, 2015) and surveys (Lu and Tsai, 2010; Fenstad et al., 2016; Sandhåland et al., 2017; Nævestad et al., 2019). Surveys are a resource-efficient way of gathering the employees’ perspectives, attitudes and experiences. It is thus possible to measure several variables simultaneously, which allows for a comprehensive analysis of the safety condition of an organisation and the relationships between various variables assumed to be related to safety. However, all self-report measures have limitations that bias the results and limit the conclusions and causal inferences. The object of the current study was therefore to investigate if factors previously identified as safety critical (see pre-print Hjellvik et al., 2019) have an impact on employees’ subsequent unwanted or dangerous behaviour.

The current study was conducted among crew on chemical tanker vessels (CTVs) operating in Arctic and Baltic waters. Work on CTVs may be characterised as hazardous with a potential for large-scale accidents that could have severe negative consequences (Arslan and Er, 2008). Work aboard the vessels may be influenced by hydrodynamics, darkness and environmental conditions such as cold or warm weather. The crew work for long hours, are away from their homes for longer periods of time, and it may be difficult to achieve proper rest and relaxation while on board. Multinational crews are common in the maritime industry, and this could impact interpersonal communication and collaboration. Further, the crew are responsible for various technical operations and monitor the condition of chemical liquids to avoid incidents such as fire, corrosion, self-reaction and poisoning (Celik, 2010). These risks have led to comprehensive regulation of work on CTVs, and requirements for competence and knowledge go beyond what is expected in other merchant fleets. Organisations with a high level of risk have a safety management system in order to monitor and react to how the safety level changes, and to enhance safety by improving relevant aspects of the sociotechnical work environment. System design, work-environment, personnel readiness and interpersonal and organisational factors should be considered in order to maintain safety on maritime vessels (Chauvin, 2011; Ding et al., 2014).

### Individual Factors That May Impact Safety

#### Safety Attitude and Reporting Attitude

An organisation’s safety culture comprises features such as values, safety perceptions, competences, behaviour and attitudes (Chauvin, 2011). Attitudes are the summarised product of affect and cognition, and attitude formation may be described as acceptance or rejection of an object (Crano and Prislin, 2006). Both organisational and interpersonal factors may impact attitudes, and norms and incentives related to safety may be prevailing elements that contribute to individual beliefs about expected safety behaviour. Ajzen (1991) suggested that attitudes, subjective norms and perceived control predict behaviour intentions with high accuracy. Cox and Cox (1991) found that safety attitudes were determined by evaluations and beliefs related to arrangements for safety, such as rules and procedures, scepticism and responsibility and risk. A more recent study from the health care sector has shown that measures of teamwork and safety climate, perceptions of management, satisfaction with work, working conditions and stress may be used to assess attitudes to safety (Sexton et al., 2006). The relationship between attitudes and behaviour is well-documented (Donald and Canter, 1994; Rundmo and Hale, 2003), and attitude to safety has been indicated to be a relevant mediator in a causal chain of safety critical factors in survey research (Hjellvik et al., 2019). This indicates that the crew’s thoughts and beliefs about risks and organisational structures impact their self-reported work performance. However, there remains a need to investigate whether attitudes predict actual behaviour.

Further, the CTV crew’s attitudes to the reporting system may influence their actual reporting behaviour. Reporting systems for incidents are often used in the maritime industry under the assumption that monitoring may highlight safety critical challenges such as system faults, human error, lack of system knowledge or excessive workload. Systematic reporting may allow for more effective and directed preventive measures. However, the reliability of reporting in the maritime industry has been shown to be of various quality (Storgård et al., 2012). Whether a given incident is reported depends on the employees’ willingness to invest time and energy in writing accurate reports when an incident occurs. It is thus reasonable that the employee’s feelings and thoughts about these systems influence their reporting behaviour and thus the frequency and quality of reports. Feedback and commitment from the management, repercussions and uncertainty about what incidents to report are some of the factors that may impact reporting motivation (see e.g., Pfeiffer et al., 2010 for a proposition of antecedents). It is thus necessary to investigate whether psychological factors such as safety and reporting attitudes of crew on maritime vessels are associated with actual safety and reporting behaviour.

#### Situation Awareness

In addition to their attitudes, the crew’s safe behaviour may also be determined by their ability to accurately detect and understand safety critical cues and anticipate further development of various scenarios that may follow given actions. The crew on CTVs have challenging work operations such as navigation, mooring, and handling and lifting of dangerous cargo. An operator needs to have an accurate online assessment of the current environment, where elements are discovered and assessed relative to other elements. This should facilitate a mental model where an operator has the necessary information to predict the potential outcomes of decisions, sometimes referred to as “situation awareness” (SA; Endsley, 1995b). Endsley described SA as a tripartite information process, where perception, understanding and prediction of a dynamic situation were represented by the term. SA reflects cognitions and knowledge about the current environment and is thus separated from both decision-making and actual behaviour. For crew on CTVs, perception of critical cues such as warning signals for the status of the content of chemical cargo are vital for further decisions of handling such cargo. This requires that the operator has sufficient attention and knowledge about the system and that the work environment provides the operator with information that is suitable for the task and the operator’s training and capacities. Accurate perception of relevant safety cues, and knowledge about the operative work environment, should provide a better basis for successful decisions that may facilitate desirable behaviour. This may thus lead to fewer dangerous incidents. Maintaining an accurate SA may be challenging as there are numerous factors that could impact an operator’s ability to identify elements, develop a coherent understanding of the relationship between them as well as anticipate the products of such outcomes. Limited attention, excessive workload and little experience with the current system are some of the factors that may reduce an operator’s ability to acquire the SA that is necessary for safe performance (Endsley, 1995b).

Various measurement approaches have been used to assess SA (Salmon et al., 2006), and SA has been found to predict performance and decision making (Strybel et al., 2008; Endsley, 2019). The concept of SA has been subject to debate, and criticism has been raised in terms of objectivism, normativism and elementarism (Dekker et al., 2010). Dekker and Hollnagel (2004) suggested a shift from the focus on cognition to a focus on the products of cognition. SA is often evaluated in terms of how well an individual’s information processing related to a given task complies with some predetermined criteria (e.g., the Situation Awareness Global Assessment Technique, SAGAT, Endsley, 1995a). Such methods can be useful to get an immediate and reliable assessment of an operator’s SA, which may provide detailed information about perception and cognition that could facilitate further improvements of sociotechnical systems. However, the work-environment aboard CTVs comprises a range of various operations and collaboration between crew members with different responsibilities. It would thus not be feasible to measure the SA of all crew members across all of their tasks. Previous studies have developed and supported an approach where employees self-report how they experience their SA across work settings (Sætrevik, 2013; Sætrevik and Hystad, 2017, 2019; Hjellvik et al., 2019). Measuring the crew’s SA, as well as other safety factors “in the field” aboard CTVs provides information about the safety level aboard the vessels and the anticipated products of the crew’s self-reported cognitive states and performance. Survey methods allow comparison between organisations, and several measurements may be used to compare levels of safety within the same organisation. It is necessary to investigate whether self-reported SA measures predict subsequent safety outcomes. It is reasonable to expect that crew who state that they have accurate SA will have a sufficient mental model for successful performance and SA should thus be associated with recorded safety events through a performance motivation that originates in successful behaviour. It is also worth noting that individual loss of SA may be identified as the cause of an incident, but this is not to say that other contextual factors that may impact the accumulated SA should be excluded from a causal analysis. Different perspectives may lead us to identify different causes for a given incident (Rasmussen, 2003), and to focus on different preventive factors.

### Safe Behaviour

The extent to which the crew on CTVs work in a safe manner is expected to be influenced by the crew’s motivation and ability to comply with the safety management system. Self-report of behaviour is often used as a proxy for actual behaviour (e.g., Sneddon et al., 2013; Sætrevik and Hystad, 2017, 2019; Hjellvik et al., 2019; Nævestad et al., 2019). Self-report of safety compliance builds on the assumption that the crew are honest in reporting their experience of their own and others’ safety behaviour, and that organisational structures facilitate adequate reporting behaviour. It also relies on the crew being able to recognise unsafe acts when they are committed or observed, and to correctly recall them in the survey. Crew that accurately report safe behaviour should contribute to a safer work environment, and these subjective statements should thus also be reflected in records registered by the organisation at a different timepoint.

#### Recorded Safety Outcomes

Heinrich’s accident triangle (Heinrich, 1931) assumed that the number of near-misses in a workplace corresponds to a lower number of minor accidents, which again corresponds to a lower number of major accidents. The aim of most safety research is to investigate factors that cause accidents, and such knowledge can be used to predict under which conditions they will occur, and thus be used to select interventions that may prevent accidents, or limit their consequences. Safety research is thus preoccupied with identifying and verifying factors that may predict observed accidents. Hofmann and Stetzer (1996) found that survey measures of safety climate and unsafe behaviour were associated with chemical processing plant accidents in the three preceding years. Although most industries have systems for monitoring and recording accidents, using major accident rates as a study’s outcome measure requires close collaboration with an industry partner, and may be difficult to interpret. The passing of time, and technological or organisational developments may lead the industry partners to be less interested in the causes of past accidents (Zohar, 2000), which may decrease the motivation for performing this kind of research. In high-reliability organisations, major accidents are rare, even when measured over time and across large organisations. It could thus be beneficial to use measures of incidents that have a more frequent occurrence (Zohar, 2000). Although the ultimate goal of safety research is to build a body of reliable knowledge that may be used to prevent major accidents, the relationship between minor and major accidents may not be as clear cut as suggested in the Heinrich’s triangle (McSween and Moran, 2017). It is thus necessary to investigate the relationship between all types of safety outcomes and specific safety behaviour.

### Research Needs and Current Study

Safety critical organisations often have systems for registration of incidents. This is typically founded on an assumption in line with Heinrich (1931) that the management of near-misses and minor accidents may prevent major accidents. The aim of the current study was to investigate if self-reported cognitions, attitudes and behaviour are associated with recorded incidents or “near-misses.” A previous analysis of self-reported variables in the current dataset (Hjellvik et al., 2019) has shown that organisational factors and safety attitudes predict safety behaviour. In that study, the self-reported statements about attitudes, cognition and safety policies were used as indicators for the crew’s self-reported safety behaviour. However, self-report of behaviour has been shown to deviate from objective measures of the behaviour (Prince et al., 2008). Self-report relies on accurate perception, introspection and recall, but accuracy is difficult to assess without additional measures. Social desirability has been argued to be a common error source in survey research (see e.g., van de Mortel, 2008, for a review), and may be described as the tendency that participants have to portray themselves in a more favourable light by exaggerating behaviour that are considered advantageous and lessen negative attributes (Edwards, 1953; Pedregon et al., 2012). It is thus necessary to investigate if measures of self-reported attitudes and behaviour are reliable and can predict recorded safety outcomes.

The current study builds on the previous analysis of the same dataset (Hjellvik et al., 2019). Using the identified relationships from that model, we add accident records in order to arrive at a coherent and clear predictive model for maritime safety. We used individual self-report of attitudes and behaviour (that were the mediators and outcomes in the previous study) as predictors of a ship-owing company’s recorded safety outcomes in the twelve months following the survey data collection. The ship-owning company’s registration of key performance indicators (KPI) of unwanted incidents were expected to have a relatively high frequency of occurrence. Individual factors such as attitudes, situation awareness and behaviour were tested against these KPIs. The first safety outcome KPI is the recording of unwanted events or acts that have been reported and categorised by the management as incidents that had the potential to trigger accidents or that was categorised as an actual accident (“Number of safety events”). The second safety outcome KPI is the recording of near misses (“Number of reports”). These are the events or conditions that have passed through one or more of the safety-barriers but were terminated before they caused an accident (Hopkins, 2009). These incidents and reports are expected to indicate the general safety level and may precede large-scale accidents. Such large-scale accidents have disastrous consequences, but for a given company or industry sector they occur so infrequently that they cannot be used as input to manage safety. The respondents in this study were employees in a Norwegian company that owns and manages CTVs operating in Arctic and Baltic waters. While safety research is often concerned with identifying risk factors for accidents, the current study employed a preventive perspective where we wanted to identify protective psychological capacities that may increase the safety of CTVs.

### Hypotheses and Pre-registration

The overview of safety research above indicates that the crew’s cognitions, attitudes and behaviour may be associated with actual safety outcomes through various psychological mechanisms. Positive attitudes to safety may indicate that the crew are motivated and able to maintain safety, which could lead to safer working practises. This leads to the following expectations: “Safety attitude” will be positively associated with “Safe behaviour” (H1a). Further, safety attitudes could lead to fewer accidents and near misses registered in the safety management system due to their motivation to perform work in accordance with the safety management system. One would thus expect “Safety attitude” to be negatively associated with “Number of safety events” (H1b). Positive attitudes may lead to improved safety and thus reduce the number of unsafe situations that the crew identify and report. However, an overly positive safety attitude may also indicate that the crew are complacent to safety issues, which may increase the number of accidents that need to be reported. We thus have a non-directional expectation of an association between “Safety attitude” and “Number of reports” (H1c).

Individuals who perceive, understand and predict safety aspects of their work environment accurately should be better able to work safely. Accurate SA may facilitate work performance and a sense of self-efficacy that may increase behaviour that is compliant with the safety management system. Employees would be motivated to recognise and respond to dangerous situations in their own and others’ work, and thus reduce the number of incidents that happen on their vessel. This leads to the expectation that “Situation awareness” will be negatively associated with “Number of safety events” (H2).

Crew members who have positive attitudes to reporting will be more willing to report a given unsafe situation that they observe. However, one may also expect positive reporting attitudes to be an expression of a safety conscious crew and a well-functioning safety management system, which leads to there being fewer unsafe situations to report. We thus have a non-directional expectation of an association between “Reporting attitude” and “Number of reports” (H3).

One may assume that crew who work safely cause fewer unsafe situations to arise, which would lead to fewer accidents on the vessel. We therefore suggest that “Safety behaviour” will be negatively associated with “Number of safety events” (H4).

We may expect that the number of employee reports of incidents correspond to actual number of incidents that occur on the vessel. A positive association could also be due to a less direct route, where the number of reports correspond to an overall safety level, which causes the number of incidents. However, there could also be a more complex relationship, where employees with better reporting attitudes are also more safety conscious, and thus cause fewer actual incidents that need to be reported, thus leading to a negative association. Our final hypothesis is thus a non-directional expectation for an association between “Number of reports” and “Number of safety events” (H5).

**FIGURE 1:**
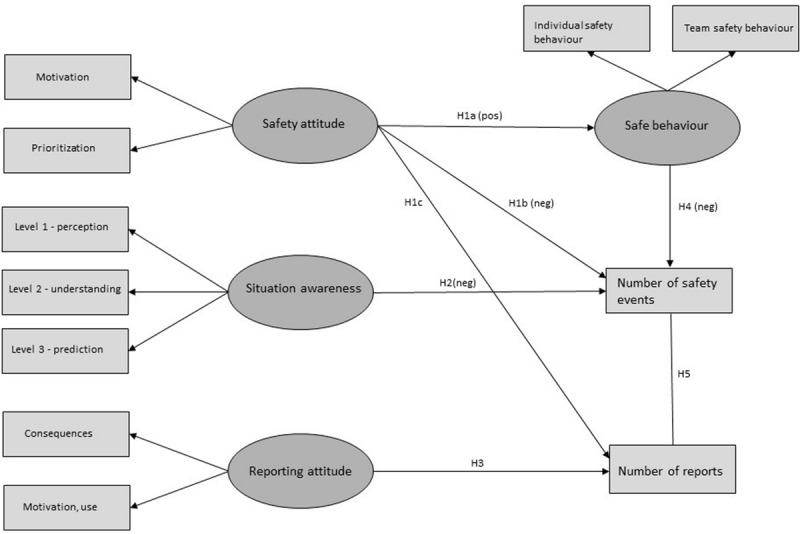
Theoretical model of the pre-registered hypotheses: H1–H5. Hypotheses H1a, H1b, H2, and H4 are marked as expecting a positive or negative association, while H1c, H3, and H5 are non-directional.

The study was pre-registered on the Open Science Framework^1^ in order to contribute to transparent research practices and the necessary research standards for reproducibility and reduction of questionable research practices. Pre-registration of studies contributes to a focus on theory development rather than significant results, and a sound theory specified *a priori* increases confidence in results independent of outcome (Van’T Veer and Giner-Sorolla, 2016). For the current study, we pre-registered the research design with the hypotheses and a complete analysis plan. The pre-registration was performed after the collection of survey data, but before collection of safety outcomes. Associations between survey variables had already been tested at the time of registration. Pre-registrations allows us to make explicit the distinction between confirmatory analyses for hypothesis testing, and more exploratory analyses that further develop the pre-registered hypotheses based on examining the collected data. The pre-registered theoretical model for the confirmatory analyses is depicted in Figure 1 above.

## Materials and Methods

### Measurement

#### Procedure

A pen-and-paper survey was sent to all employees working on CTVs in a Norwegian ship-owning company. The company transports chemical cargo in the Arctic and Baltic waters. The surveys were coded to identify responses from the same vessel, and to be able to associate them with subsequent safety outcomes. The ship-owning company’s health, safety, environment and quality (HSEQ) department distributed the surveys to the vessels. The ship-owning company held the code key to associate vessel name with survey code, but the ship-owning company did not receive the completed surveys or dataset. This system ensured that the researchers could not know the name of vessels or their crew, and the ship-owning company could not know the responses of crew members or specific vessels. The manning on the vessels is organised in shifts, and surveys were distributed to approximately 450 employees. The participants did not receive payment for participation, but they were encouraged to participate, and received one reminder to complete the survey. The survey had 122 statements about organisational, interpersonal and individual safety factors. The statements asked for demographic information and the extent to which the participants agreed to statements about safety issues such as the accuracy of their situation awareness, the captain’s and ship-owning company’s ability and willingness to focus on safety, their own safety behaviour and use of safety tools, and the extent to which they express positive safety attitudes. The statements were rated on a 5-point Likert-style scale from “Completely disagree” (1) to “Completely agree” (5). Some of the items were semantically reversed so that a score on “Completely agree” would count against the calculated variable. An overview of all items used in the survey is available online^2^, showing which items were reversed and how they assemble to variables and sub-factors.

The data for the outcome variables were registered and collected in the 12 months after the survey data collection was completed. The ship-owning company’s safety management system registered the number of reports and incidents that occurred on the CTVs from April 2018 to April 2019. A total of 1540 reports were registered during this period, which corresponds to 4.2 unwanted events per day across the 18 vessels. The data was registered for each vessel code and sent to the researchers to be added to the survey data set. Each crew member was assigned the number of incidents and “near miss” registered to their vessel. To control for the reduced variability in the dataset that this approach led to, analyses were performed with clustering for vessel.

#### Participants

Three hundred and twenty eight surveys were returned to the researchers (73% of the distributed surveys). Fifteen surveys were removed from the sample due to apparent language issues (e.g., ignoring all reversed items) and blank or identical answers on more than 33% of the items. In accordance with the pre-registered analysis, 21 captains were also excluded, resulting in a sample of 292 participants.

The participants worked on deck (59%), in the engine room (31%) and in the galley (10%). There were 11–28 participants from each of the 18 vessels, all male. The participants were primarily from the Philippines (75%) and Latvia (17%), but participants from Russia, Norway, Lithuania and “Other” were also represented. The majority stated that they had worked for the ship-owning company for 5 years or more (60%), but 49% stated that they had worked on the current vessel for less than a year. This indicates that the crew alternate between working on various vessels in the company’s fleet. The first page of the survey provided consent information, and participants were informed that the survey was voluntary and anonymous. The project was evaluated by The Norwegian Centre for Research Data (project 56912), prescribing guidelines for the handling of personal data.

#### Variables

“Situation awareness” was measured with 13 statements that describe the perception, comprehension, and assessment of a situation. The items were developed by Sætrevik (2013) and represent Endsley’s theoretical model (Endsley, 1995b) where SA has a tripartite division. Items thus represent the extent to which the participant tends to perceive safety-critical cues in their working-situation (level 1), understand contingencies between them (level 2) and predict how safety aspects of the situation will develop over time (level 3). The three levels are sub-levels of SA and level 1 was measured with four items, level 2 was measured with five items and level 3 was measured with four items. A sample item is: “*I notice when an unsafe situation is about to arise at my workplace.”* Higher score on the variable indicates that the participant has accurate SA across various relevant work-settings. Five of the items were semantically reversed in order to prevent response biases. Raykov’s reliability coefficient for SA was 0.78. This is above 0.7 and thus satisfactory for the cut-off of scale reliability for being used in confirmatory factor analysis (CFA) and structural equation modelling (SEM; Mehmetoglu and Jakobsen, 2017).

“Safety attitude” was measured with 13 statements that describe the beliefs, feelings and thoughts the participants may have about safety. The items represent the participant’s motivation to follow safety procedures and how they prioritise safety in their day-to-day work. The two subcategories “motivation” and “prioritisation” were measured with nine and four items, respectively. Some of the statements were developed by the researchers and subject matter experts, some of the items were inspired by the ship-owning company’s safety campaign and some of the items were from scales developed by Rundmo (1994) and Nielsen et al. (2013). An example of a negatively phrased item is: *“Safety procedures often stand in the way of getting the job done efficiently.”* Higher score on the variable indicates that the participant has a positive attitude to safety aboard the vessels. Four items were semantically reversed. Raykov’s reliability coefficient was 0.56 and thus not satisfying the preferred criteria of 0.7, which may indicate that the items do not represent a unified concept for this sample.

“Reporting attitude” was measured with 12 statements that describe the beliefs, feelings and thoughts the participants may have about the company’s reporting system for accidents and “near-misses”. The item texts used both terms (as “accidents/near-miss”) since the ship-owning company tended to use them in concert in their safety communication with the crew. The scale comprises items related to assumed consequences of reporting, and motivation to use the reporting system and actual use of the reporting system. The subcategories “consequences” and “motivation and use” were measured by eight and three items, respectively. Some of the items were inspired by Probst and Graso (2013) and were supplemented by additional items developed through iterative biennial surveys for a different maritime sector (see pre-registration Sætrevik, 2017), as well as through discussions with HSEQ officers for the current industry partner. A higher score on the variable indicates that the participant has positive attitudes to reporting unwanted incidents. Ten items were semantically reversed, and an example of a negatively phrased item is: *“Reporting all accidents/near-misses will not be helpful to increase safety.”* Raykov’s reliability coefficient was 0.83.

“Safe behaviour” was measured with seven statements that describe actions that the crew may perform during their work that has positive or negative impact on safety. Some of the items were from the “Brief safety climate inventory” (Nielsen et al., 2013) and supplemented with items developed in collaboration with industry experts to suit the current setting. Four of the items describe how the respondent collaborate with other crew members to maintain safety (team safety behaviour), while three of the items describe how the respondent’s own actions are relevant for safety (individual safety behaviour). Higher score on the variable indicates that the participant follows the safety procedures and expectations in their work. Five of the items were semantically reversed. An example of a negatively phrased item is: *“I sometimes expose myself or others to danger in order to get the job done.”* Raykov’s reliability coefficient was 0.71.

“Safety KPIs” were recorded by the ship-owning company as part of their regular system for monitoring vessel safety by asking crew to report all unwanted incidents and “near-misses.” All reports from the vessels were collected in the 12 months after the surveys had been returned to the researchers. Each report was classified by the company as either constituting an actual incident or a “near-miss.” Both categories originate from the reports filed by the crew and safety officers that were later classified into the categories by the management based on severity. The number of reports classified as “near-miss” per vessel were used as the “Number of reports” variable for all crew members assigned to that vessel. The remaining reports for the vessel were used as the “Number of incidents” for the crew members on that vessel. While the pre-registration suggested an additional follow-up analysis where incidents were classified according to severity, examining the recorded KPIs and discussing it with our contacts in the ship-owning company revealed that this was not feasible. In compliance with our pre-registered analysis plan, no outliers were removed from the sample.

### Analyses

**FIGURE 2:**
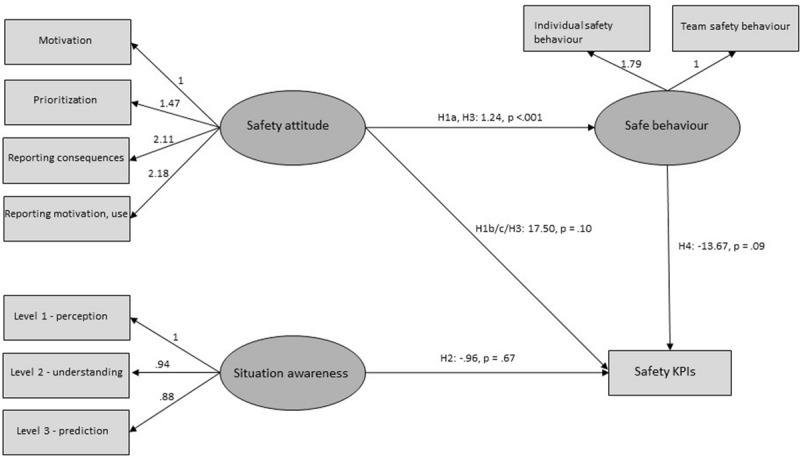
Results from the exploratory SEM-analysis where “Reporting attitude” and “Safety attitude” are merged into one variable, and the total number of reported incidents are combined in one outcome variable. The figure shows unstandardised coefficients and two-tailed *p*-values, marked with hypothesis enumeration from the pre-registration. Please note that the pre-registration described that the non-directional hypotheses were to be tested with two-tailed tests and the directional hypotheses were to be tested with one-tailed tests. For simplicity, only two-tailed *p*-values are displayed in the figure but results from the respective tests are presented in the text. Error terms and observed indicators are hidden in the figure.

Structural equation modelling (SEM) was used to test the pre-registered hypotheses of associations between subjective and objective factors (using the gsem module in STATA). The pre-registered confirmatory structural model for testing the hypotheses is shown in Figure 1. In addition, an exploratory structural model was tested after reviewing the preliminary analyses and results of the confirmatory model (shown in Figure 2).

## Results

### Model Testing

There was a correlation of *r* = 0.76 between “Safety attitude” and “SA,” 0.86 between “Safety attitude” and “Safe behaviour” and 0.68 between “SA” and “Safe behaviour.” “Safety attitude” and “Reporting attitude” were separate variables in the pre-registered model but were correlated by 0.96 and may thus be theoretically overlapping. These variables were therefore merged into one “Safety attitude” variable in the exploratory model (see below).

The pre-registered structural model tested the associations between the crew’s self-reported statements about safety outcomes with their vessel’s actual performance. Results from testing the pre-registered model with and without clustering (see details in the online Supplemental Materials^3^) did not support any of the hypotheses. However, an inspection and discussion with the industry partners revealed that the safety KPIs had been measured somewhat differently from the description in the pre-registration. “Number of safety events” and “Number of reports” were initially expected to be from separate reporting systems, but had in fact originated from the same reports, with a more ambiguous distinction between the two categories. This reduces our confidence that the pre-registered model is the best approach to use the current dataset to answer the pre-registered research questions. Thus, a follow-up exploratory structural model was tested to address this limitation while answering the pre-registered hypotheses H1-H4 (thus excluding H5). The exploratory model is a simplification of the pre-registered model, where “Reporting attitude” and “Safety attitude” are merged into “Safety attitude,” and the two types of KPIs are collapsed (see Figure 2 above). Results with clustering showed SRMR = 0.058 for the CFA and SRMR = 0.06 for the SEM. The pre-registration described that the directed hypotheses were to be tested with one-tailed tests, and the non-directional hypotheses were to be tested with two-tailed tests. Full results and fit indexes for the analysis performed without clustering are available in the Supplementary Material online^4^.

#### Support for H1 and H3

The positive association between “Safety attitude” and “Safe behaviour” (corresponding to H1a and H3 in the pre-registered model) has previously been supported in the current dataset (although previous test did not include reporting attitude items). We therefore do not consider the test to be confirmatory in the current analysis but include it in order to make the current models coherent and to compare this association with the others in the model. H1a and H3 constitute the same association in the exploratory model, and was found to be supported (with two-tailed testing). The association indicates that those of the crew who have positive safety and reporting attitudes also perform their work safely.

The exploratory model did not show a significant association between “Safety attitude” and the total number of KPIs (H1’, corresponding to H1b/c and H3 in the pre-registered model, two-tailed tests were applied as no clear directional hypothesis can be extracted from the pre-registration). This indicates that the crew’s reported attitudes to the safety aspects of their work did not impact the number of reports or incidents from their vessel. However, please note that a positive association was significant with one-tailed testing.

#### Support for H2

“SA” was not significantly associated with the total number of KPIs in the exploratory model (H2’, one-tailed testing in accordance with the pre-registration). This indicates that the measure for crewmembers’ accuracy of the crew’s perception, comprehension and prediction of the safety aspects of their work did not predict the number of reports or incidents from their vessel.

#### Support for H4

“Safe behaviour” was negatively associated with the total number of KPIs in the exploratory model (H4’, significant with one-tailed testing in accordance with the pre-registration). This indicates that there are fewer incidents on vessels with crew members that state that they follow the safety procedures in their work.

## Discussion

### Summary of Results

It has been suggested in the scientific literature that various individual factors influence safety-critical behaviour. These factors are typically measured by self-report but are assumed to impact the objective risk of accidents in the workplace. The present study aimed to test whether self-reported individual factors influence subsequent recorded safety outcomes. Data were collected among the crew employed in a ship-owning company that transports chemical cargo. The crew’s self-report of “Safe behaviour” was associated with the ship-owning company’s KPIs in the exploratory model, but the remaining hypotheses and the pre-registered model were not supported.

### Association of Survey Measures and Objective Outcomes

#### Prediction of Safety Outcomes

As shown in a previous analysis of the self-repoted measures in this dataset (Hjellvik et al., 2019), “Safety attitude” was associated with “Safe behaviour” (H1a). This indicates that crew who state that they have positive attitudes to safety also state that they follow procedures and regulations in their work. This association was included in the current analysis model in order to complete the model and allow comparison with the other associations. The remaining associations (H1b – H5) were not supported in the pre-registered model. This indicates that we were not able to predict the independent effects of the crew’s evaluation of their attitudes, SA and behaviour aboard the vessels on their vessel’s safety outcomes in the following year.

Due to uncertainty regarding how the company measured the KPIs, and in order to better answer some of our pre-registered hypotheses, we additionally tested an exploratory model of the pre-registered hypotheses between survey responses and safety outcomes. Our initial and pre-registered expectation of the ship-owning company’s reporting system did not correspond to how the system was actually used. As all safety outcomes are reports of unwanted incidents, we found it necessary to collapse both KPIs to a total number of safety outcomes independent of the management’s *post hoc* categorisation of the reports. Further, the two variables “Reporting attitude” and “Safety attitude” were highly correlated. Although there are conceptual differences between what the two constructs were intended to measure, the correlation indicated that the constructs were not differentiated in the current sample, and the variables were therefore merged.

To compensate for the uncertainty in how events were coded and that an arbitrary division of event reports may hide associations, we modified the pre-registered model to an exploratory model where both event types were collapsed into one observed outcome variable. The explorative model showed a negative association between “Safe behaviour” and the combined safety outcomes. This indicates that, in line with the H4 expectation, the crew members that stated that they work safely had fewer accidents and reports of unwanted incidents on their vessels in the subsequent year. Such a mechanism requires that (1) Crew members can assess and report their safety behaviour, (2) The behaviour has an impact on the safety of the vessel, and (3) The ship-owning company’s KPIs are indicators of the level of safety on the vessels. There are few comparable studies in the maritime safety literature, but several studies conducted in other industries have found associations between self-report and registered injuries and accidents (Zohar, 2000; Glasscock et al., 2006; Zadow et al., 2017).

#### Limitations With the Survey Measure

The other predictors, “SA” and “Safety attitude” were not associated with safety outcomes. As discussed above, previous research has associated SA for a given task with performance on that task (see e.g., Endsley, 2019). It is therefore reasonable to assume that crew members who report that individual and environmental factors allow them to perceive relevant safety elements (level 1 of SA), understand how they are connected (level 2 of SA), and anticipate what the composition of elements could lead to (level 3 of SA) will also perform their work more safely. If a context-general self-report measure is related to aspects of SA and determines the extent to which crew are able to work safely, one may expect it to predict safety outcomes for the vessel. However, limitations with the survey design may have obscured relationships between variables. The current inventory of SA measures the participants’ assessment of their SA across work settings and is thus an assessment of how participants think they handle safety in their work in general. This is in contrast to most SA measures, which are related to a specific task with clear evaluation criteria. This difference in measurement approach may be biased by the participants’ knowledge, motivation and memory of the safety of their work actions.

We expected individuals who reported positive attitudes to safety management and reporting system to work more safely. Previous research has shown that attitudes are related to behaviour (see e.g., Ajzen, 1991), and attitudes have been shown to partially mediate the relationship between safety climate and safety behaviour (Clarke, 2010). The relationships have been claimed to be stronger when specific attitudes relate to specific actions (Ajzen and Fishbein, 1973). This could indicate that reporting attitudes have higher predictive value than safety attitudes, as deciding whether to report a given event or not has a close correspondence to an attitude, while safety attitudes may correspond to several different decisions or actions. As mentioned above, social desirability bias may have contributed to obscure a relationship between attitudes and subsequent safety outcomes in the current study. When using self-report measurement of how the crew prioritises safety, there may be biases to conform to the company’s expected safety standard. Although we collaborated with the ship-owning company’s personnel department to adapt the survey to be suited for crew with English as a second language, language issues may still have detracted from an accurate measure of attitudes. A prior analysis of the current dataset (Hjellvik et al., 2019) revealed that the negatively phrased items had lower average scores than the positively phrased items, which may indicate that the items were not always correctly interpreted. Such factors may have prevented relationships from being identified.

#### Limitations With the Safety Outcomes

The lack of significant associations between survey measures and safety outcomes may also be due to the company’s KPIs not being reliable measures of the actual safety level. Reports of incidents are assumed to provide knowledge about the level of safety in the workplace, are used to monitor the frequency of undesirable events and to decide how to manage safety (Reiman and Pietikäinen, 2012). However, as KPIs are reported by the crew and management, various factors may influence how well individuals are able to and are motivated to notice, reflect upon and report an incident accurately^5^. Some organisations set targets for the number of reports the workforce is expected to submit, which may lead to a focus on managing the measure (Hopkins, 2009), and the crew may become preoccupied with “reporting for the sake of reporting.” Macrae (2016) highlighted several problems with reporting systems in health care, and stated that reports may be biased by cognitive, social and organisational factors. For instance, various factors could increase reports of some incidents and decrease reports of other, which would detract the reliability of the KPIs as measures of actual safety.

#### Limitations With the Concept of Reporting

As described in the Introduction, reporting may have a complex association with actual safety, which makes it challenging to predict the direction of the relationships. More reports of accidents or near-misses could indicate that an unsafe work environment has led to more dangerous situations, but it could also indicate that a more safety-conscious crew are able to recognise risks and motivated to report them. It is possible that both types of mechanisms were active in our setting, which could have cancelled out or confounded any systematic variation between the variables. If vessel or company management formally or informally encourage the vessels to submit a minimum number of reports, it is difficult to know how such encouragement will be interpreted by the crew, and how it affects the reliability of the reports.

### Implications and Further Research

The literature reviewed above has shown that various individual and organisational factors may influence safety behaviour. In the current study, self-reported safety behaviour predicted subsequent safety outcomes, as individuals that work safely have fewer “near-misses” and incidents. This indicates that large-scale surveys where employees report their safety-behaviour may be valuable for safety monitoring. Safety management systems should therefore take organisational, individual, interpersonal and socio-technical factors into consideration through multidisciplinary top-down system approaches (Rasmussen, 1997; Yemao et al., 2018). Survey studies are not useful to investigate all of the factors in complex systems, but may provide information about specific safety critical aspects. While there will always be variability in human performance, a safety management system that results in employees that are motivated, willing and able to work safely will facilitate beneficial safety outcomes.

While we found support for a relationship between subjective and objective measures when applying an exploratory SEM model, it should be noted that our pre-registered model which distinguished between events and “near-misses” was not supported. This demonstrates how tenuous these relationships may be and emphasises the importance of being able to predict associations with pre-registered hypotheses and analysis methods in order to claim causal relationships. The exploration of data should be clearly delineated from the confirmatory analyses in order to identify relationships that can be reliably reproduced and form a robust basis for safety management policies.

Further studies should continue the investigation of associations between self-reported safety factors and actual unwanted incidents in the maritime industry. The relationship between reporting and accidents needs to be further explored. While this issue is complicated to resolve, it is no less important, since so much safety management research and practice in the maritime industry relies on the assumed relationship between reporting and safety. We suggest that further research on this issue could use reporting attitude measures that are supplemented by other approaches. A possible approach could be to present brief descriptions of hypothetical low-risk situations and ask employees how likely it is that they would report such a case. It could also be beneficial to investigate various other measures of attitudes and cognitive states with actual safety behaviour. Further studies could benefit from including scales that measure biases (such as social desirability) along with measures of subjective and objective data. In addition, a longitudinal design could investigate predictors and outcomes over time. Such designs may provide more reliable information about causal relationships and would provide a stronger foundation for safety management.

## Conclusion

Reliable empirical knowledge about causal relationships is needed to maintain safety in the maritime industry. The current study builds on the overall assumption that cognition, attitudes and behaviour predict subsequent safety outcomes on maritime vessels. The results showed an association between self-report of safety behaviour and objective safety outcomes, but there were no associations between the crew’s safety attitudes and objective safety outcomes, or between SA and objective safety outcomes. The variables measured are often mentioned as “safety critical,” but the current study highlights the importance of designing studies where we can be more certain of the reliability and validity of results. A substantial part of safety research literature in the maritime industry consists of associations between self-reported measures, either cross-sectional or longitudinally, and most of this research does not have a pre-registered transparent process with objective outcomes. Without establishing the external validity of the measures and the relationship to actual safety outcomes, and without being able to control for undeclared exploration of data, it is difficult to say how much confidence we should have in such findings. The current study suggests that such issues should be emphasised in future research.

## Data Availability Statement

The survey, dataset, models and pre-registration can be found online at: https://osf.io/qfcv3/.

## Ethics Statement

The studies involving human participants were reviewed and approved by The Norwegian Centre for Research Data. The patients/participants provided their written informed consent to participate in this study.

## Author Contributions

BS designed the theoretical model and initiated the data collection. LH was the leading author. LH and BS approved the final manuscript.

## Conflict of Interest

The authors declare that the research was conducted in the absence of any commercial or financial relationships that could be construed as a potential conflict of interest.

## Footnotes

1 https://osf.io/cj8fr

2 https://osf.io/sfpu5/

3 https://osf.io/ks2xr/

4 https://osf.io/eq9uk/

5 We hypothesised that “near-miss” reports would be more influenced by attitudes while events would be more influenced by behaviour, and that it could be informative to examine the relationship between the two types of KPIs. However, since none of the associations were significant, and we are unsure whether the two types of KPIs could be distinctly categorised, it is difficult to draw any conclusions on this matter.
